# Impact of Protein Energy Malnutrition on Outcomes of Adults With Viral Pneumonia: A Nationwide Retrospective Analysis

**DOI:** 10.7759/cureus.12274

**Published:** 2020-12-25

**Authors:** Valeria P Trelles-Garcia, Daniela Trelles-Garcia, Asim Kichloo, Sairam Raghavan, Pius E Ojemolon, Precious Eseaton, Osahon N Idolor

**Affiliations:** 1 Internal Medicine, John H. Stroger, Jr. Hospital of Cook County, Chicago, USA; 2 Internal Medicine, Saint Francis Hospital, Evanston, USA; 3 Internal Medicine, Central Michigan University, Saginaw, USA; 4 Internal Medicine, John H Stroger, Jr. Hospital of Cook County, Chicago, USA; 5 Anatomical Sciences, St. George's University, St. George's, GRD; 6 Internal Medicine, College of Medicine, University of Benin, Benin City, NGA

**Keywords:** viral pneumonia, protein energy malnutrition, nis, mortality, morbidity

## Abstract

Background

Community-acquired pneumonia (CAP) is associated with significant morbidity and mortality. Viral organisms have been identified as the causal pathogen in approximately 20% of CAP. Nutritional status plays an important role in the response to pneumonia. This study aims to identify whether protein energy malnutrition (PEM) is an independent risk factor for mortality and morbidity in viral CAP.

Materials and methods

This was a retrospective cohort study involving adult hospitalizations for viral CAP in the United States using the Nationwide Inpatient Sample (NIS) database. This cohort was further divided based on the presence or absence of a secondary discharge diagnosis of PEM. The primary outcome was inpatient mortality. Secondary outcomes included the rate of mechanical ventilation among other complications.

Results

The in-hospital mortality for viral CAP was 2.22%. Patients with PEM had over two-fold high adjusted odds of inpatient mortality (aOR: 2.42, 95% CI: 1.746-3.351, p < 0.001) compared with patients without PEM. Patients with PEM had higher adjusted odds of having septic shock (aOR: 3.34, 95% CI: 2.158-5.160, p < 0.001). NSTEMI (aOR: 1.75, 95% CI: 1.163-2.621, p = 0.007), need for mechanical ventilation (aOR: 3.13, 95% CI: 2.448-4.006, p < 0.001), CVA (aOR: 3.49, 95% CI: 1.687-7.220, p = 0.001), DVT (aOR: 2.19, 95% CI: 1.453-3.295, p < 0.001), and PE (aOR: 2.24, 95% CI: 1.152-4.357, p = 0.017) relative to patients without PEM.

Conclusion

In conclusion, coexisting PEM is associated with a higher rate of in-hospital morbidity and mortality in patients with viral CAP. Early identification and treatment of nutritional deficiencies can lead to improved outcomes and reduced costs.

## Introduction

Community-acquired pneumonia (CAP) ranks first in hospitalizations and seventh in all-cause death in industrialized countries [1]. Viral organisms have been identified as the causal pathogen in approximately 1/5th of CAP. About 100 million cases of viral CAP occur every year among adults, with a mortality rate estimated to be between 3% and 24% [2,3]. Viral pathogens that cause CAP include influenza, adenovirus, parainfluenza, respiratory syncytial virus, and human metapneumovirus. Advanced age, smoking, obesity and environmental exposures to different substances including metals, dust, and fumes have been linked to worse outcomes in patients with viral CAP [3,4].

Nutritional status plays an important role in the outcome of many disease conditions [5-8]. Nutrition is postulated to be involved in the immune response to pneumonia. Protein energy malnutrition (PEM) is reportedly linked to lymphoid tissue atrophy, decreased delayed cutaneous hypersensitivity, fewer T cells, impaired secretory immunoglobulin A antibody response, decreased antibody affinity, reduced concentration and activity of complement components and phagocyte dysfunction [9]. Some studies found that PEM is associated with increased morbidity and mortality, longer length of hospital stay, and increased hospital costs [10,11] in patients with viral CAP. Moreover, it was found to be an independent predictor of 2-year mortality after discharge in the elderly patients [12]. However, the relatively small sample sizes of these studies raise concern for sample bias. The purpose of this study is to identify whether PEM is an independent risk factor for mortality and morbidity in viral CAP using population-level data.

## Materials and methods

Design and data source

This was a retrospective cohort study for viral CAP in the United States between January 1, 2016 and December 31, 2017. The Nationwide Inpatient Sample (NIS) databases for 2016 and 2017 were used. The NIS is a database of hospital inpatient stays derived from billing data submitted by hospitals to statewide data organizations across the United States, covering more than 97% of the U.S. population [13]. It approximates a 20% stratified sample of discharges from U.S. community hospitals, excluding rehabilitation and long-term acute care hospitals. This dataset is weighted to obtain national estimates [14]. Both the 2016 and 2017 databases were coded using the International Classification of Diseases, Tenth Revision, Clinical Modification/Procedure Coding System (ICD-10-CM/PCS).

Study population

Adult patients 18 years and above who had a principal discharge diagnosis of viral CAP including influenza due to identified novel influenza A virus with pneumonia (J09.X1), influenza due to other identified influenza virus with pneumonia (J10.0), influenza due to unidentified influenza virus with pneumonia (J11.0), and viral pneumonia, not elsewhere classified (J12) were included. Patients were excluded if they had super-imposed bacterial pneumonia or if viral pneumonia was only a secondary diagnosis. This cohort was further divided based on the presence or absence of a secondary discharge diagnosis of PEM (E43, E44.0, E44.1, E46).

Outcome measures

The primary outcome was comparing inpatient mortality among hospitalizations principally for viral CAP between PEM and non-PEM patients. Secondary outcomes in this population included odds of having a secondary discharge diagnosis of sepsis, septic shock, acute respiratory failure, acute respiratory distress syndrome, Non-ST segment elevation myocardial infarction (NSTEMI), acute kidney failure (AKI), deep vein thrombosis (DVT), pulmonary embolism (PE), cerebrovascular accident, need for mechanical ventilation, vasopressors as well as mean length of hospitalization (LOS) and mean total hospital charges (THC).

Statistical analysis

We analyzed the data using Stata ® Version 16 software (StataCorp, College Station, TX). All analyses were conducted using the weighted samples for national estimates in adjunct with Healthcare Cost and Utilization Project regulations for using the NIS database. Co-morbidities were calculated as proportions of the cohort and Chi-squared test was used to compare these characteristics between subgroups. Multivariate regression analysis was done to adjust for possible confounders while calculating the primary and secondary outcomes. This method was employed in prior peer-reviewed publications [15-18]. Possible confounders were obtained from the literature review, and included variables from the validated MuLBSTA Score for viral pneumonia as well as the Pneumonia Severity Index [19,20]. A univariate screen was done to further confirm possible confounders with variables having a p < 0.2 included in the multivariate regression analysis. A p of 0.05 was set as the threshold for statistical significance.

Ethical considerations

The NIS database lacks patient-level identifiers. Since 2012, the NIS has also removed most state level and hospital identifiers. This study was exempt from Institutional Review Board approval.

## Results

Patient characteristics

The combined NIS database for 2016 and 2017 contained over 71 million weighted hospital discharges of which 89,650 satisfied the inclusion criteria for the study. These hospitalizations were for adult patients with a principal discharge diagnosis of viral CAP. Of these patients, 5.0% had a secondary diagnosis of PEM.

Table 1 details the patient and hospital characteristics of the studied cohort. Patients with PEM were significantly older (71.1 vs 67.8 years, p < 0.001). and were more likely to have comorbid history of malignancy (58.4 vs 37.4%, p < 0.001), anemia (43.2 vs 23.9%, p < 0.001), chronic obstructive pulmonary disease (32.1 vs 25.4%, p < 0.001) and chronic kidney disease (20.2 vs 16.6%, p = 0.006) compared to patients without PEM. However, patients with PEM had significantly less proportion of co-morbid hypertension (34.7 vs 41.7%, p < 0.001) and diabetes (24.5 vs 31.9%, p < 0.001). There was no significant difference in past or current use of tobacco between the subgroups.

**Table 1:** Patient and hospital characteristics of hospitalizations for viral pneumonia by PEM. #: for 2017. CHF: congestive heart failure, CKD: chronic kidney disease, COPD: chronic obstructive pulmonary disease, CVA: cerebrovascular accident, IHD: ischemic heart disease, PEM: protein energy malnutrition, US$: United States dollars.

Variable	Overall	Non-PEM %	PEM %	p-value
	89,650	n = 85170 (95.0)	n = 4480 (5.0)	
Patient characteristics				
Age, mean		67.8	71.1	<0.001
Female	55.1	55.1	53.7	0.416
Racial distribution				<0.001
White	68.2	68.1	69.3	
Black	11.4	11.5	9.5	
Hispanic	9.9	10.0	5.0	
Others	10.5	10.4	13.2	
Insurance type				<0.001
Medicaid	66.9	66.4	75.4	
Medicare	10.0	10.0	10.2	
Private	20.1	20.5	12.7	
Uninsured	3.0	3.1	1.7	
Charlson Comorbidity Index score				<0.001
0	19.1	19.5	12.3	
1	27.1	27.2	24.9	
2	19.2	19.2	19.7	
≥3	35.6	34.1	43.1	
Median annual income in patient’s zip code, US$^#^				0.577
1-43,999	28.6	28.5	29.4	
44,000-55,999	25.5	25.5	24.6	
56,000-73,999	24.5	24.6	23.2	
≥74,000	21.4	21.4	22.9	
Co-morbidities				
Diabetes melltus type 1 & 2	31.5	31.9	24.5	<0.001
Hypertension	41.3	41.7	34.7	<0.001
Smoking history	37.9	37.9	38.6	0.671
CHF	22.8	22.6	26.7	0.004
CKD	16.8	16.6	20.2	0.006
Dyslipidemia	39.2	39.5	33.8	<0.001
Anemia	24.3	23.3	43.2	<0.001
Chronic IHD	23.8	23.9	23.4	0.781
Prior CVA	2.2	2.1	2.9	0.133
Malignancy	38.4	37.4	58.4	<0.001
COPD	25.7	25.4	32.1	<0.001
Oxygen dependent	6.7	6.5	8.9	0.005
Hospital characteristics				
Hospital region				0.002
Northeast	18.7	18.8	17.2	
Midwest	25.0	24.7	30.1	
South	35.3	35.5	30.1	
West	21.0	21.0	22.6	
Hospital bed size				0.002
Small	22.8	22.9	20.0	
Medium	27.8	27.9	24.4	
Large	49.4	49.2	55.6	
Urban location	87.7	87.4	93.0	<0.001
Teaching hospital	62.3	62.0	69.2	<0.001

Primary outcome: in-hospital mortality

The in-hospital mortality for patients principally hospitalized for viral pneumonia was 2.22%. Patients with PEM had over two-fold high adjusted odds of inpatient mortality (aOR: 2.42, 95% CI: 1.746-3.351, p < 0.001) compared with patients without PEM.

Secondary outcomes

Patients with PEM had higher adjusted odds of having septic shock (aOR: 3.34, 95% CI: 2.158-5.160, p < 0.001), NSTEMI (aOR: 1.75, 95% CI: 1.163-2.621, p = 0.007), need for mechanical ventilation (aOR: 3.13, 95% CI: 2.448-4.006, p < 0.001), CVA (aOR: 3.49, 95% CI: 1.687-7.220, p = 0.001), DVT (aOR: 2.19, 95% CI: 1.453-3.295, p < 0.001), and PE (aOR: 2.24, 95% CI: 1.152-4.357, p = 0.017) relative to patients without PEM. Patients with PEM also had significantly longer LOS as well as higher THC. Table 2 provides a summary of the clinical outcomes.

**Table 2:** Clinical outcomes of hospitalizations for viral pneumonia by PEM. *Statistically significant, ^#^adjusted mean difference. aOR: adjusted odds ratio, CI: confidence interval, ARDS: acute respiratory distress syndrome, NSTEMI; non-ST segment elevation myocardial infarction, PEM: protein energy malnutrition, US$: United States dollars.

Outcome	Non-PEM, %	PEM, %	aOR (95% CI)	p-value*
Primary outcome				
In hospital mortality	2.0	6.3	2.42 (1.746 – 3.351)	<0.001*
Secondary outcomes				
Length of stay, mean	4.9	9.1	3.63^ # ^(2.987 – 4.277)	<0.001*
Total hospital charges, mean US$	43995	93841	42591^# ^(30879 - 54304)	<0.001*
Sepsis	2.9	8.3	2.58 (1.962 – 3.387)	<0.001*
Septic shock	0.8	3.6	3.34 (2.158 – 5.160)	<0.001*
NSTEMI	1.7	4.0	1.75 (1.163 – 2.621)	0.007*
Mechanically ventilated	3.5	11.2	3.13 (2.448 – 4.006)	<0.001*
Required pressors	2.2	2.8	1.14 (0.746 – 1.734)	0.542
Acute kidney failure	17.2	25.9	1.45 (1.220 – 1.723)	<0.001*
Acute respiratory failure	25.1	32.6	1.33 (1.139 – 1.544)	<0.001*
ARDS	0.8	1.6	1.78 (0.990 – 3.188)	0.054
Deep vein thrombosis	1.1	3.4	2.19 (1.453 – 3.295)	<0.001*
Pulmonary embolism	0.5	1.2	2.24 (1.152 – 4.357)	0.017*
Cerebrovascular accident	0.3	1.2	3.49 (1.687 – 7.220)	0.001*

## Discussion

About 5% of adults with viral pneumonia had a secondary diagnosis of PEM. In-patient mortality was two times more likely to occur if viral pneumonia coexisted with PEM. These patients also had high odds of requiring mechanical ventilation, having septic shock, DVT, PE, had longer hospital stay, and incur higher hospital costs compared to patients without PEM.

Only 5% of patients with viral CAP had PEM, which was significantly lower than previously reported. One study showed that the prevalence of malnutrition is as high as 40% among hospitalized patients [21], rising to 53.4% of elderly patients [12]. The low prevalence of PEM in the study population may be explained by under-diagnosis; the lack of an initial nutritional assessment in many hospitalized patients may be the driving factor. The absence of a standardized definition of PEM is another contributor. Hypoalbuminemia, hypoproteinemia, malnourishment, among others could have been used as surrogate diagnosis for PEM. The increased risk of septic shock is probably related to the detrimental effect that malnutrition has in the immune response. however, the relation between nutritional status and deep venous thrombosis or pulmonary emboli is unclear. Decreased ambulatory capacity in the setting of poor nutrition and critical illness remains a hypothesis. The association between longer length of hospitalization with higher hospital charges and PEM is well documented. This has a remarkable implication for healthcare-related costs. If malnutrition was optimally assessed, and treated, CAP economic burden could decrease significantly. Nutritional status is a proven determinant of the health trajectory and outcome following hospital discharge [12]. Yeo et al showed that despite the resolution of pneumonia, malnourished elderly people have an increased risk of future impaired muscle and respiratory function, leading to increased long-term mortality [12]. Mortality increased in proportion to the degree of malnutrition [22]. We agree that targeted risk reduction interventions based on recognizing risk factors for CAP are of primary importance in reducing CAP related death rates [23]. This study emphasizes the need to assess every patient’s nutritional status on admission, particularly in the elderly. It is worth mentioning that to date, no single parameter is definitive for determining malnutrition in the elderly [24].

This study has some important limitations. The NIS uses "claims data", and ICD-10 codes were used to confirm diagnoses rather than clinical parameters [25,26]. NIS does not appropriately grade disease severity [26]. It involved a retrospective database subject to nonrandomization. Microbiologic confirmation of specific viral causes of CAP is cost-intensive with little clinical relevance, hence often not performed routinely. Sputum and blood culture-negative bacterial CAP, especially atypical CAP may be misclassified as viral CAP. NIS report data hospitalization data, rather than individual patients, hence patients with multiple hospital encounters will be captured multiple times [27,28]. NIS does not contain information on disease duration [29], hence we cannot determine if this PEM disease duration may have played a role in the outcomes of viral pneumonia hospitalizations. NIS studies do not prove causation, only association can be obtained. Nonetheless, this database provided the unique opportunity to study this cohort at a population level. Multiple centers are accounted for ensuring representation of an ample geographic area. Comprehensive literature search, use of sound methodological review techniques and an exhaustive review process have developed this piece of evidence. 

## Conclusions

In conclusion, coexisting PEM is associated with higher rate of in hospital morbidity and mortality in patients with viral CAP. Early identification and treatment of nutritional deficiencies can lead to improved outcomes and reduced costs.

## References

[1] Tokgoz Akyil F, Yalcinsoy M, Hazar A (2018). Prognosis of hospitalized patients with community-acquired pneumonia. Pulmonology.

[2] Ruuskanen O, Lahti E, Jennings LC, Murdoch DR (2011). Viral pneumonia. Lancet.

[3] Almirall J, Serra-Prat M, Bolíbar I, Balasso V (2017). Risk factors for community-acquired pneumonia in adults: a systematic review of observational studies. Respiration.

[4] Shaka H, Raghavan S, Trelles-Garcia VP (2020). Predicting COVID-19 using retrospective data: impact of obesity on outcomes of adult patients with viral pneumonia. Cureus.

[5] Shaka H, Edigin E, Raghavan S (2020). The obesity paradox among patients hospitalized for bacterial pneumonia: outcomes of the nationwide inpatient sample. Chest.

[6] Shaka H, Ojemolon PE (2020;12(10). Impact of obesity on outcomes of patients with hip osteoarthritis who underwent hip arthroplasty. Cureus.

[7] Shaka H, Gomez TM (2020). Bronchogenic malignancies and the obesity paradox: adding weight to a growing argument using the nationwide inpatient sample. Chest.

[8] Shaka H, Padilla Sorto ME, Gomez TMA, Edigin E, Xu J, Yap SET (2020). 242-LB: the obesity paradox among patients hospitalized for diabetes and its complications: outcomes of the nationwide inpatient sample. Diabetes.

[9] Chandra RK (1991). Protein-energy malnutrition and immunological responses. J Nutr.

[10] Barker LA, Gout BS, Crowe TC (2011). Hospital malnutrition: prevalence, identification and impact on patients and the healthcare system. Int J Environ Res Public Health.

[11] Jensen GL (2006). Inflammation as the key interface of the medical and nutrition universes: a provocative examination of the future of clinical nutrition and medicine. JPEN J Parenter Enteral Nutr.

[12] Yeo HJ, Byun KS, Han J (2019). Prognostic significance of malnutrition for long-term mortality in community-acquired pneumonia: a propensity score matched analysis. Korean J Intern Med.

[13] Edigin E, Ojemolon PE, Eseaton PO (2020). Systemic sclerosis is associated with increased inpatient mortality in patients admitted for atrial fibrillation: analysis of the national inpatient sample [ahead of print]. J Clin Rheumatol.

[14] Edigin E, Ojemolon PE, Eseaton PO (2020). Rheumatoid arthritis patients have better outcomes when hospitalized for ischemic stroke: analysis of the national inpatient sample [ahead of print]. J Clin Rheumatol.

[15] Ojemolon PE, Shaka H, Edigin E (2020). Impact of diabetes mellitus on outcomes of patients with knee osteoarthritis who underwent knee arthroplasty: an analysis of the nationwide inpatient sample. Cureus.

[16] Edigin E, Shaka H, Eseaton P (2020). Rheumatoid arthritis is not associated with increased inpatient mortality in patients admitted for acute coronary syndrome. Cureus.

[17] Asemota I, Shaka H, Ehizogie E (2020). TCT CONNECT-78 coexisting protein energy malnutrition is associated with increased mortality in patients admitted for transcatheter aortic valve replacement: analysis of the national inpatient sample. J Am Coll Cardiol.

[18] Shaka H, Raghavan S, Edigin E (2020). Is the presence of diabetes mellitus a poor prognostic factor during hospitalizations for bacterial pneumonia? An analysis of the nationwide inpatient sample. Chest.

[19] Guo L, Wei D, Zhang X (2019). Clinical features predicting mortality risk in patients with viral pneumonia: the MuLBSTA score. Front Microbiol.

[20] Fine MJ, Auble TE, Yealy DM (1997). A prediction rule to identify low-risk patients with community-acquired pneumonia. N Engl J Med.

[21] Kopelman P, Lennard-Jones J (2002). Nutrition and patients: a doctor's responsibility. Clin Med.

[22] Rodríguez-Pecci MS, Carlson D, Montero-Tinnirello J, Parodi RL, Montero A, Greca AA (2010). Nutritional status and mortality in community acquired pneumonia [Article in Spanish]. Medicina.

[23] Almirall J, Bolíbar I, Serra-Prat M (2008). New evidence of risk factors for community-acquired pneumonia: a population-based study. Eur Respir J.

[24] Harris D, Haboubi N (2005). Malnutrition screening in the elderly population. J R Soc Med.

[25] Jamal S., Khan M.Z., Kichloo A. (2021). The effect of atrial fibrillation on inpatient outcomes of patients with acute pancreatitis: a two-year national inpatient sample database study. J Innov Cardiac Rhythm Manage.

[26] Edigin E, Akuna E, Asemota I (2020). Rheumatoid arthritis does not negatively impact outcomes of patients admitted for atrial fibrillation. Cureus.

[27] Edigin E, Eseaton P, Kaul S (2020). Systemic sclerosis is not associated with worse outcomes of patients admitted for ischemic stroke: analysis of the national inpatient sample. Cureus.

[28] Edigin E, Kaul S, Eseaton PO (2020). Analysis of hidradenitis suppurativa hospitalizations: a report from the National Inpatient Sample database (in press). J Am Acad Dermatol.

[29] Edigin E, Ojemolon PE, Eseaton PO (2020). Systemic sclerosis is associated with increased inpatient mortality in patients admitted for acute coronary syndrome: analysis of the national inpatient sample (ahead of print). J Clin Rheumatol.

